# Preparedness through social capital in Bengawan Solo River Communities, Surakarta City

**DOI:** 10.4102/jamba.v17i1.1857

**Published:** 2025-09-18

**Authors:** Yanis R. Ningtias, Rita Noviani, Pipit Wijayanti, Afzal A. Osza

**Affiliations:** 1 Department of Geography Education, Faculty of Teacher Training and Education, Universitas Sebelas Maret, Surakarta, Indonesia

**Keywords:** Bengawan Solo River, floods, ordinary least squares, preparedness, social capital, Surakarta city

## Abstract

The city of Surakarta is prone to flooding because of the flow of the Bengawan Solo River, particularly in densely populated areas along its banks. Key contributing factors include inadequate drainage systems, illegal development, climate change, irregular spatial planning and low public awareness of disaster risks. This study investigates the role of social capital in enhancing community preparedness for flood disasters along the banks of the Bengawan Solo River. The quantitative approach employed a survey method using a Likert scale questionnaire ranging from 1 to 5, distributed to 87 respondents from 29 affected communities. Data analysis was conducted using multiple regression with the ordinary least squares (OLS) method. The results indicated a positive but not significant relationship between exposure to flood risk and social capital (coefficient = 0.624769; *p* = 0.267555), suggesting that increased exposure did not significantly correlate with strengthened social capital. Nevertheless, these findings highlight the importance of social capital in enhancing community preparedness and mitigating the negative impacts of floods.

**Contribution:**

Strategic recommendations include strengthening social networks and trust among citizens, conducting disaster simulations and training, implementing early warning systems, restructuring flood-prone settlements, improving infrastructure, creating green open spaces and promoting sustainable data-driven disaster education. These efforts are expected to enhance community resilience in addressing flood disasters in a participatory and sustainable manner.

## Introduction

Flooding is a major challenge for many cities in developing countries, particularly in tropical regions with high rainfall. Land use changes, rapid urbanisation and environmental degradation exacerbate the impact of flooding in areas where drainage systems cannot handle rainwater runoff (Hapsoro & Buchori 2015). In Indonesia, floods are the most common hydrometeorological disasters, causing significant damage to infrastructure, economic losses and threats to human life, especially in urban areas.

One of the cities that faces serious challenges from flood disasters is the city of Surakarta. The city has a high level of vulnerability because it is crossed by the Bengawan Solo River, the longest river on the island of Java, with a watershed area of about 16 100 km^2^ (Ningsih & Dewi 2024). History records that major floods because of the overflow of this river have occurred several times, including in 1918, 1966, 2007, 2009 and 2023 (Fariza, Hasim & Rahadianto 2016; Lasminto et al. 2016). The Indonesian government, through the 2020–2024 Medium-Term Development Plan (RPJMN), has even set the restoration of the Bengawan Solo watershed as a national priority because of its condition, which is considered critical.

Densely populated settlements on the banks of the Bengawan Solo River are the areas most affected by river overflows, especially in the rainy season. The high intensity of rainfall, combined with inadequate river capacity, a lack of green open spaces and insufficient drainage systems, leads to inundation spreading into residential areas (Araujo, Astuti & Yudana 2023; Ningrum 2020). Settlements in this area generally consist of buildings with low construction quality that are not designed to be flood-resistant and are inhabited by people with limited socio-economic conditions (Noviani, Sarwono & Muryani 2021). This condition results in significant exposure to flood risk, both physically and socially (Setyaningrum, Rahmawati & Marfai 2019).

On the other hand, the potential of communities in building resilience to disasters is often overlooked in flood management approaches. Social capital, such as trust between citizens, community networks and the spirit of mutual cooperation, is an important asset in strengthening the adaptive capacity of communities against disaster risk (Restu Meyda et al. 2023). Social capital can play a key role in improving communication, accelerating the distribution of aid and devising more effective evacuation strategies (Norzistya & Handayani 2020). However, in practice, this social dimension has not been widely accommodated in disaster risk mitigation policies and programmes at the local level. Previous research has tended to focus on physical and technical aspects, without examining the social potential of communities in the context of disasters (Noviani 2022).

Based on these conditions, it is important to conduct a study highlighting social capital’s role in increasing preparedness and reducing community exposure in densely populated areas along the banks of the Bengawan Solo River. This study aims to explore the role of social capital in enhancing community capacity to address flood disasters, while also examining the relationship between preparedness and levels of flood exposure. The findings are expected to contribute to the development of more effective, contextual and sustainable community-based mitigation strategies that align with the local social characteristics of Surakarta.

## Literature review

### Population density and regional vulnerability

The densely populated settlement on the banks of the Bengawan Solo River in Surakarta City is a representation of the complexity of the relationship between spatial pressures because of urbanisation, physical and social vulnerability and the adaptive capacity of communities to flood disasters. The population density in this area is inseparable from the limited land in the core area of the city, the high accessibility to the centre of economic activities and the low economic level of the community, which forces them to look for settlements at low costs. In this context, Ramadhan, Hadiani and Muttaqien (2024) highlight that the riverbank area is an attraction for low-income people because of its relatively low cost of living and strategic location. However, the area is also known as a highly vulnerable area to flooding, making it not ideal as a residential zone.

Various studies have shown that the high population density on the banks of rivers magnifies the risk of flooding, especially when accompanied by the presence of household and business infrastructure that is not designed to deal with hydrometeorological disasters. Absori et al. (2023) and Lasminto et al. (2016) emphasise that uncontrolled settlement and economic activities exacerbate the region’s exposure to flooding. This problem is compounded by poor drainage systems, the existence of illegal buildings, land use changes and low public awareness of disaster risks (Taryana et al. 2022). If left unchecked, this condition not only increases the frequency and intensity of floods but also increases physical vulnerability, especially in buildings that do not have disaster resilience standards.

### Physical and social vulnerability to flooding

Vulnerability to flooding in this region can be classified into two main dimensions, namely physical and social vulnerability (Arif, Mardianta & Giyarsih 2017; Pontoh, Sangkertadi & Tilaar 2021; Rachmat 2014; Urbanus, Sela & Tungka 2021). From a physical point of view, the quality of building construction plays a vital role in determining the extent to which a household can withstand waterlogging (Yamin et al. 2022). It was stated that building structures made of durable materials, such as bricks, are more resistant to water pressure than buildings made of lightweight materials, such as wood. In addition, older buildings tend not to have implemented flood-resistant engineering standards, in contrast to newer constructions that are beginning to adopt adaptive building principles. The location factor also influences buildings that are too close to the riverbank, which have a higher risk of significant flooding (Farid et al. 2020).

Social vulnerability also appears significantly in the communities living in this region. Low-cost settlements, although more economically affordable, are generally inhabited by community groups with limited resources. Murtiono et al. (2020) and Rachmayani (2023) emphasise that this group of communities is more vulnerable to the impact of floods because of limited access to information, adaptive infrastructure and social support. When floods hit, they experience difficulties in evacuation, asset rescue and post-disaster recovery. This combination of physical and social vulnerability makes the Bengawan Solo River area a high-risk zone that requires community-based mitigative interventions.

### The importance of social capital in disaster mitigation

Social capital is one of the essential factors in disaster management (Norzistya & Handayani 2020). Social capital at the community level and its social networks are an important part of disaster management efforts (Norzistya & Handayani 2020). In recent years, social capital has begun to be recognised as an important element in increasing people’s capacity to face disasters (Norzistya & Handayani 2020). This demonstrates that social trust, networks of relationships among citizens and collective participation are essential factors that enhance community resilience. This social capital facilitates effective communication, the rapid dissemination of evacuation information and the exchange of resources during crisis situations (Jafari 2024; Susanto & Kusumasari 2023). Reinforcing this argument by emphasising that communities that have a high level of trust between members are better prepared to respond to disasters. In fact, they are more actively involved in disaster simulations, emergency response training and self-help contingency planning (Abunyewah et al. 2023). Unfortunately, this dimension often goes unnoticed by policymakers who focus more on technical and infrastructure solutions in disaster mitigation.

### The urgency of improving community-based preparedness

The urgency to strengthen community-based preparedness is becoming increasingly important, especially in the context of densely populated areas such as those on the banks of the Bengawan River in Solo (Absori et al. 2023). It is noted that the evacuation process is a major challenge when floods hit densely populated areas because of the complexity of logistics and the diversity of social conditions of the population. In addition, the differences in vulnerability that each individual or community has, both from demographic, psychological and spatial aspects (Yamin et al. 2022), demand an adaptive and participatory approach that integrates local knowledge and the social power of communities. Noviani et al. (2021) emphasised that the preparedness of people in flood-prone areas can be significantly improved through strengthening their social capital.

Thus, this study takes a strategic position to fill gaps in the literature that still lack a comprehensive integration of four key dimensions – population density, physical vulnerability, social vulnerability and social capital – in understanding and improving flood preparedness. This research also targets a specific local context, namely the Bengawan Solo coastal area, which has not been studied in depth in the global literature. The approach used aims not only to identify vulnerability and preparedness levels but also to develop community strength-based strategies. This novelty is expected to be able to make a substantive contribution, both at the academic level and in community-based disaster policy.

## Research methods and design

### Research areas

This research was conducted in a densely populated residential area located along the Bantran River, Bengawan Solo, which is administratively included in the area of Surakarta City, Central Java Province. The research location includes 29 communities (Rukun Warga, or RW) spread across two sub-districts, namely Pasar Kliwon District (including Mojo, Semanggi, Sangkrah and Sewu Villages) and Jebres District (including Pucangsawit and Jebres Villages).

**FIGURE 1:**
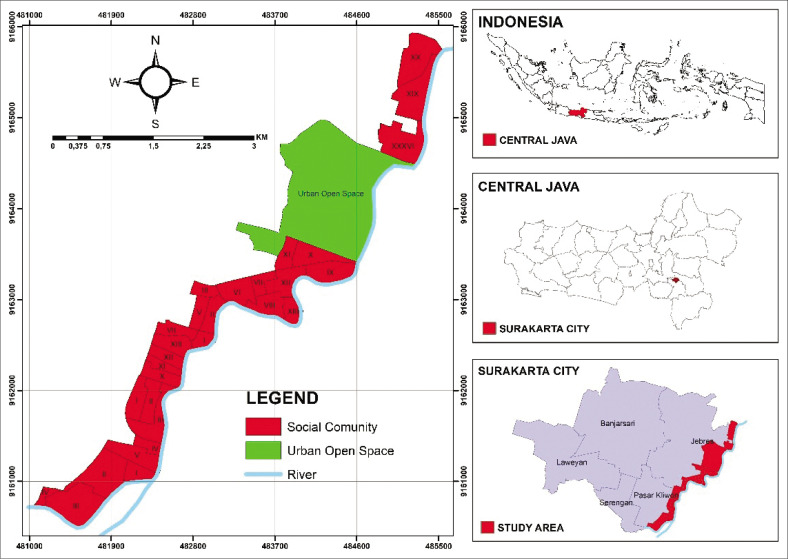
Study area of densely populated settlements along the Bengawan River in Solo, Surakarta city.

The selection of this area was based on the results of preliminary surveys that showed the dominance of densely populated settlements on the banks of the river. This area is known to have high vulnerability to flooding because of its proximity to river flows and lack of flood management infrastructure. Figure 1 shows the location of the study in the geographical context of Indonesia.

The Bengawan Solo River flows through several major cities in Central Java Province, including Surakarta. This main river and its tributaries significantly contribute to recurrent flooding in the densely populated areas surrounding them (Farid et al. 2020; Ramadhan et al. 2024). The high rate of urbanisation in the city of Surakarta, especially in riverbank areas that have relatively low land prices, encourages the growth of many new settlements (Wibawa, Utomo & Miladan 2022). These characteristics also increase the risk of flooding (Ramadhan et al. 2024). The large number of informal settlements and slums causes buildings to not meet technical standards, and supporting facilities such as drainage systems and flood management are inadequate. This condition exacerbates the vulnerability of the population during floods (Rentschler & Salhab 2020).

The green open space (Urban Open Space), such as city parks and zoos in the area, was not included in the scope of the study because they were not inhabited by the community.

### Sampling procedures and methods

**FIGURE 2:**
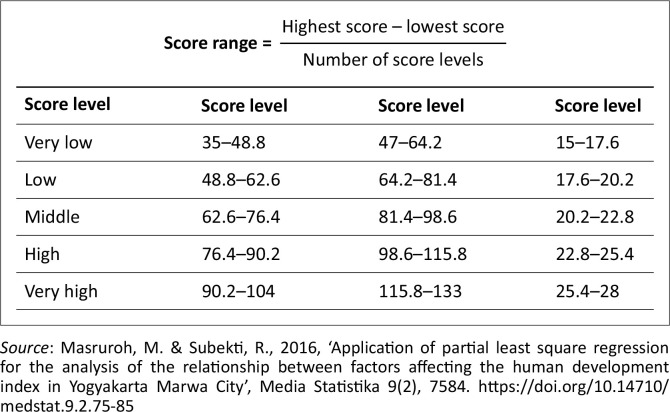
Classification of Likert scale variables.

A quantitative approach was used in this study, using an instrument in the form of a questionnaire on a Likert scale of 1–5. Respondents were asked to provide an assessment of statements related to three main dimensions: social capital, flood preparedness and flood exposure. The Likert scale used includes values from 1 (strongly disagree) to 5 (strongly agree). The classification of variables and assessments is shown in Figure 2.

**FIGURE 3:**
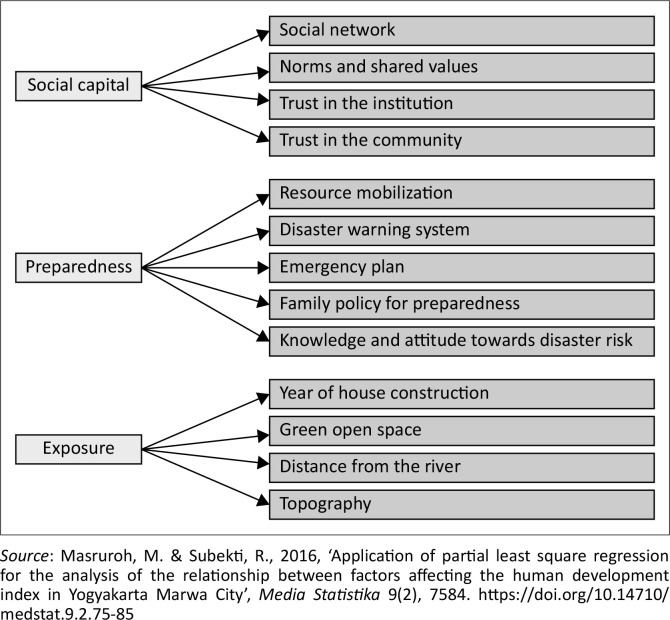
Variables used in the study.

The selection of variables is based on relevant literature studies and adjusted to empirical conditions in the field. The conceptual framework and variables used in this study are presented in Figure 3.

The research sample was taken from people living in flood-prone areas along the Bengawan Solo River, Surakarta City. The sampling technique used is stratified random sampling to ensure the representation of each flood risk zone. Each community is divided into three zones based on proximity to the river and its level of risk:

1. Edge zone (high risk): Directly adjacent to rivers and most prone to flooding.
2. Middle zone (medium risk): Somewhat far from a river with medium vulnerability.
3. Upper zone (low risk): Farthest from the river and lowest risk.

From each zone, one respondent was randomly selected, so that three respondents from each community were obtained. The total number of respondents was 87 people from 29 communities. This approach allows for a comprehensive picture of people’s perceptions, preparedness and vulnerability to flood disasters.

### Data analysis

The survey results were analysed using the multiple linear regression technique with the ordinary least squares (OLS) method (Sari 2021) (see Table 1). The regression model is used to test the relationship between independent variables (e.g. dimensions of social capital) and dependent variables (e.g. flood preparedness and exposure) (Masruroh & Subekti 2016). This analysis aims to identify the extent to which social factors play a role in shaping community preparedness and the level of exposure to flooding in densely populated residential areas along the Bengawan River in Solo, Surakarta.

**TABLE 1:** Summary of ordinary least squares result model variables.

Variable	Coefficient [a]	SE	*t*-statistic	Probability [b]	Robust SE	Robust *t*	Robust Pr [b]	Lively [c]
Intercept	13.316351	13.218804	1.007379	0.323038	9.661485	1.378292	0.179858	-
Preparedness	0.658699	0.091612	7.190055	0.000000*	0.124900	5.273823	0.000016*	1.005996
Exposure	0.624769	0.551429	1.133000	0.267555	0.376677	1.658633	0.109205	1.005996

Note: The asterisk (*) is used to mark statistically significant variables, namely variables that have a *p*-value below the significance threshold, generally 0.05.

SE, standard error; Pr, probability.

### Ethical considerations

Ethical clearance to conduct this study was obtained from the Faculty of Teacher Training and Education Research Ethics Committee, Universitas Sebelas Maret (reference no.: 07/UN27.02.11/PP/EC/2024).

## Results and discussion

The primary purpose of this study is to determine the role of social capital of densely populated communities on the banks of the Bengawan Solo River, Surakarta City, which is also related to their preparedness and exposure when facing flood disasters. In this study, 87 respondents provided information related to their social capital, preparedness and exposure, with the following respondent characteristics.

**FIGURE 4:**
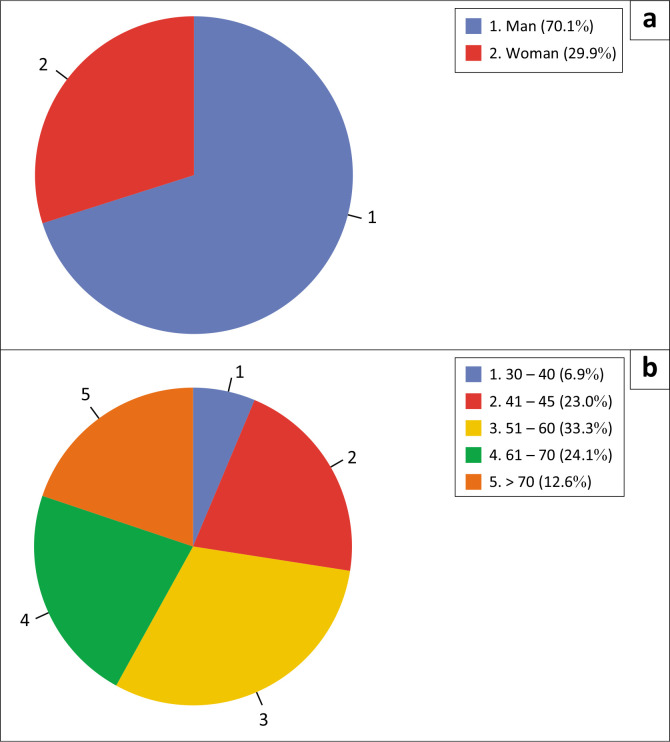
Characteristics of the respondents’ (a) gender and (b) age.

Figure 4 indicates that 70.1% of respondents were male and 29.9% were female, with the predominant age range being 51 to 60 years. All respondents were indigenous peoples from the study area, with an average length of residency exceeding 30 years. Additionally, all respondents reported experiencing the impact of the flood disaster on the Bengawan Solo River.

**FIGURE 5:**
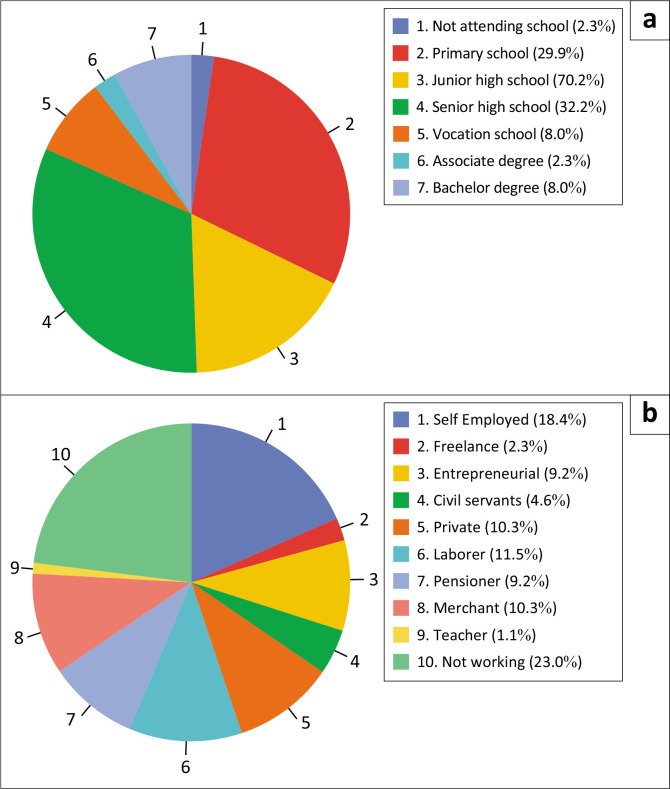
Distribution of respondents’ education and employment levels (a) gender and (b) age.

In Figure 5, it can be seen that most of the respondents have primary to secondary education. Primary to secondary education levels can affect awareness and understanding of flood disasters (Primaditya et al. 2024). People with basic education usually have limitations in understanding technical information about disaster mitigation or how to reduce risk (Septikasari 2022). Conversely, this study indicates that social capital, including mutual cooperation and strong social relationships, is prevalent in communities with lower levels of secondary education and can enhance community-based preparedness. Respondents primarily work in the informal sector as traders, laborers, or self-employed individuals, with some not currently employed. This group often faces economic instability, which complicates their ability to financially prepare for flood mitigation strategies (Arif et al. 2017). Low education and informal employment increase people’s vulnerability to flood impacts, especially in terms of exposure and access to better infrastructure. Ability to adapt or recover after a flood becomes more limited for this group (Arif et al. 2017). This condition is in line with the findings of researchers in the research area.

Many low-income workers live in suburbs or underdeveloped areas where disaster management infrastructure and facilities may be inadequate, making them more vulnerable to flood risks. However, those who actively engage with their communities can participate more effectively in preparedness training and simulation programmes. Building substantial social capital can help low-income workers mitigate the impact of flood disasters. Community building that strengthens social networks and improves access to information can improve preparedness and reduce exposure. Communities along the Bengawan River in Solo, Surakarta City, which are dominated by junior secondary education and jobs dominated by the informal sector, have low preparedness, as seen in Figure 5.

**FIGURE 6:**
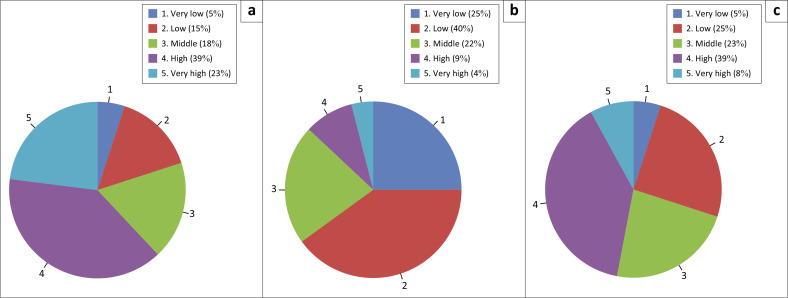
Respondent interview results (a) social capital, (b) preparedness and (c) exposure.

Figure 6 shows that 39% of people are in the high exposure category and 8% are in the very high category. This shows that about 47% of the population is exposed to a high risk of flooding. This high level of exposure is because of the geographical location along the river, which is very vulnerable to flooding, especially when there is an increase in water discharge in the Bengawan Solo River. This condition is caused by inadequate infrastructure, such as slum conditions, poor drainage, minimal vegetation space and poor embankment function. The average distance from respondents’ houses to the river ranges from less than 500 m to a maximum of 2 km. Many respondents live along the banks under river embankments, which pose significant dangers and are at risk of flooding during rainfall. Additionally, settlements in the study area are predominantly comprised of buildings over 20 years old, increasing the potential for flood damage and highlighting the inadequacy of irrigation canals.

Figure 6 shows that most people are in the low 40% and very low 25% exposure categories. The findings of this study show that most of the communities that have low preparedness do not reflect less than optimal preparedness in dealing with floods. However, they are not ready because people are used to flood disasters. Flood disasters are an annual occurrence for the community, and many residents have come to accept them. This acceptance often leads to a lack of preparedness; people do not take precautions because they are accustomed to flooding and simply evacuate when a disaster strikes. Most of the communities in the study area explained that they had no procedures in place during the floods. People only rely on their spontaneity when a flood disaster occurs. Most people also do not have emergency measures to deal with floods because they are used to dealing with other disasters. Most people also never take part in training and simulations in flood management. Evacuation routes are also very minimal in every area of the community. The community considers that they no longer need an evacuation route because they are used to the route and the place. The absence of clear procedures shows that the community does not have a systematic preparedness plan. In the research area, there are still very few early warning systems, and some flood detection devices are damaged. This condition requires the community to detect flood disasters manually through weather monitoring and looking at river discharges. In some communities, some people communicate with the government and sluice gate officers to monitor the arrival of floods.

Despite having low preparedness and high exposure, the community along the Bengawan River in Solo, Surakarta City, has high social capital. As many as 39% of communities are at a high level of exposure to flood disasters, and 23% are at a very high level. This shows that most people in the research area have strong social capital. High social capital is crucial during disasters, as it indicates strong social networks and collective cooperation among citizens, which can be leveraged during or after a crisis. The high power of social capital can be used to deal with low preparedness and high exposure. With strong social relationships, people can support each other in various social, economic and health aspects. This condition can help strengthen community preparedness and be ready to face the threat of flooding and reduce its impact. The power of social bonds can be harnessed to enhance collective cooperation during disasters. People between communities also often exchange information related to flood disasters. Most people also trust their community leaders when providing information related to flood disasters.

Most people consider mutual cooperation essential in their community. Such collaboration fosters a sense of solidarity among members. When a flood disaster strikes, working together can strengthen social ties and enhance trust among individuals. This creates an environment where every community feels a responsibility to help each other, both in preparation before floods, during evacuations and in the post-disaster recovery process. Mutual cooperation also allows for more effective management of resources. In times of disaster, people can support each other through energy, foodstuffs or equipment. By sharing resources, they can meet basic needs during and after floods, thus speeding up the recovery process. Through mutual cooperation activities, the community can increase awareness of flood risks and the importance of preparedness. Most people also help each other in terms of health by helping each other and providing medicines. Communities also monitor and patrol when their homes are flooded to monitor water discharge and ensure the safety of their homes. This activity involves all family members with a specific division of tasks.

A positive and significant relationship (*p* = 0.000000) is indicated by a coefficient of 0.658699, which shows that preparedness significantly affects social capital. This suggests that as community preparedness increases, social capital will also rise. The *p* of 0.000000 further confirms that this relationship is statistically significant, providing strong evidence that consistently improving preparedness will enhance social capital in the region.

Social capital plays a major role in building community preparedness for flood disasters. Social capital includes trust, social networks and cooperation in society. People with high social capital, such as strong trust between citizens and involvement in social activities, tend to be better prepared for disasters. It is easier for them to work together in disaster mitigation activities, such as cleaning waterways, sharing information on early warnings and evacuating together when floods occur.

Based on the findings of research by the community along the Bengawan River in the city of Surakarta, most of them explained that they trust their neighbours during floods and help each other socially, physically and healthily. The community also exchanged information related to the flood. The community is collaborating to address flooding in their area because they care about one another. However, they have all been affected by the flood disaster. These findings indicate that preparedness plays a crucial role in strengthening social capital among residents. When individuals engage in joint preparedness activities such as evacuation exercises, trust and social collaboration are formed, which has an impact on strengthening social capital (Abunyewah et al. 2023). This is consistent with the findings of Abunyewah et al. (2023), which states that people with strong social networks are better prepared to respond to disasters.

However, these results differ from the findings of Restu Meyda et al. (2023) and Budhiana and Dewi (2023). This shows that high social capital is always followed by high preparedness. In the context of the community on the banks of the Bengawan Solo River, low preparedness is accompanied by high social capital, which allows the community to survive even with the limitations of the formal system.

Meanwhile, high exposure does not show a significant effect on social capital. Despite this, communities living in flood-prone zones show a tendency to form social groups and networks as survival mechanisms. Social capital also plays a role in encouraging community collaboration to build flood management infrastructure collectively and in demanding support from the government.

Preparedness fosters trust among members of society, which is a key component of social capital (Abunyewah et al. 2023). When individuals engage in collective preparedness activities, such as exercises and planning sessions, they build relationships that increase mutual support during disasters (Manna et al. 2023). People who have a strong social network will be better equipped to gather and take action based on important information during a flood (Rustinsyah, Azis & Adib 2021). This condition can have a positive impact on overcoming the low preparedness of densely populated residential communities along the Bengawan Solo River. Despite having low preparedness, the community has high social capital to help each other during flood disasters. The community also often carries out post-flood handling activities, such as cleaning the environment and setting up emergency tents.

The presence of social capital enables communities to mobilise resources and coordinate responses effectively. Preparedness activities foster collaboration among citizens, local organisations and government agencies, leading to a more organised and efficient response to flood events (Rustinsyah et al. 2021). Although there is no formal preparedness or specialised training, high social capital allows communities to act quickly when floods occur. Communities rely on local information from community leaders and nearby neighbours to take joint action. High social capital means that people have great trust in their community. Social capital reflects the existence of strong social bonds among people. This can be seen in the culture of cooperation during disasters, where communities help each other by providing temporary shelter, food or physical energy to help neighbours who are more severely affected by the floods. This solidarity can accelerate post-disaster recovery. When floods hit, communities rely on internal support systems, such as fundraising, the provision of temporary shelter and the provision of basic resources such as food, water and clothing. This assistance is carried out spontaneously by community members who have close relationships with each other.

In a society with high social capital, the flow of communication runs faster because trust between community members is high. It is easier for communities to organise to evacuate, provide resources or keep the area safe from further risks during flooding. Although there is no formal protocol, this spontaneous action allows for a more effective response. High social capital allows for the formation of informal aid networks that can be mobilised immediately during floods. This network involves family, friends and neighbours helping each other with resources or energy. This social capital can be used to improve community preparedness. Disaster education programmes can be carried out collectively, leveraging social relationships built to disseminate mitigation information and establish better early warning mechanisms. Although communities around rivers may not have adequate technical preparedness, their high social capital can be a strong foundation for improving community-based disaster response and enhancing long-term resilience.

The relationship between flood risk exposure and social capital is positive but not significant, with a coefficient of 0.624769 and a *p* of 0.267555. This suggests that while increased exposure to flood risk is associated with an increase in social capital, the association is not statistically significant as the *p* exceeds the threshold of 0.05. In summary, although there is a tendency for greater exposure to correlate with higher social capital, this relationship lacks the strength needed to be deemed statistically significant.

People who live on the banks of rivers or in low-lying areas are more exposed to the risk of flooding. When rainfall is high or rivers overflow, the risk to communities in these areas increases significantly. People living in high-risk areas tend to be more aware of the threat of flooding, prompting them to build stronger social networks as mitigation efforts. Many people are involved in community groups that focus on disaster preparedness and response. People tend to be more involved in building strong social networks in areas with high flood risk. Social capital can serve as a mitigation tool in overcoming exposure. By having a solid network, people can more quickly access information, share resources and take necessary precautions.

Social capital also plays a role in reducing people’s exposure to floods. This exposure includes factors such as proximity to the river, topography and the condition of buildings and green open spaces. Communities with strong social capital can work together to build or improve flood control infrastructure, such as strengthening embankments, repairing drainage channels or regreening vulnerable areas. People with high social capital tend to be more proactive in encouraging the government to make the necessary interventions to reduce exposure. In communities along the Bengawan Solo River, social capital, preparedness and exposure are three variables that affect each other in dealing with flood risk. Strong social capital promotes better preparedness and, at the same time, helps reduce the negative impacts of high exposure. On the other hand, the impact of high physical exposure can be minimised if the community has good preparedness and strong social capital. Therefore, a holistic approach that integrates social capital enhancement, preparedness and physical exposure mitigation is essential to increase community resilience to flooding along the Bengawan Solo River.

In a study conducted by Ndlovu and Msimanga (2015), social capital is used to increase drought resilience. The involvement of various socio-economic groups is important in shaping and realising community welfare. Cooperation between various socio-economic groups highlights the value of capital interdependence and the interrelated effects of resource utilisation on building resilience (Ndlovu & Msimanga 2015). The integration of social capital enhancement, technical preparedness and physical risk mitigation must be a strategic approach to building community resilience. Ndlovu and Msimanga (2015) also found that social capital serves as a crucial foundation for long-term resilience, though it still requires support from external sources, such as the government and humanitarian institutions.

## Conclusion

This study reveals that densely populated areas along the banks of the Bengawan Solo River in Surakarta City are highly vulnerable to flood disasters. Specifically, 39% of respondents fall into the high exposure category, and 8% into the very high category, indicating that nearly half of the population resides in a serious flood risk zone. Conversely, community preparedness remains low, with 40% of respondents classified as having low preparedness and 25% as very low preparedness.

Nevertheless, the people in the study area possess a relatively strong level of social capital. Specifically, 39% of the community has a high level of social capital, while 23% have a very high level. High social capital is reflected in the strong social network, the spirit of mutual cooperation, trust between citizens and collective support in dealing with disasters. This social capital functions as an important basic capital in increasing community resilience, especially in conditions of limited formal infrastructure.

The results of the regression analysis showed that there was a positive and significant relationship between preparedness and social capital (*p* = 0.000000). This means that increasing community preparedness can significantly strengthen their social capital. Conversely, although exposure also showed a positive relationship with social capital (coefficient = 0.624769), the relationship was not statistically significant (*p* = 0.267555). This suggests that although people in high-exposure areas tend to have stronger social capital, this tendency has not been statistically confirmed in the context of this study.

In general, there are interdependent interactions between the three main variables – social capital, preparedness and exposure – in shaping community resilience to flood risk. Strong social capital can drive better preparedness and, at the same time, help mitigate the negative impacts of high exposure. Conversely, high exposure can be more easily dealt with if people have good preparedness and strong social networks.

### Practical recommendations

Several strategic steps can be taken to enhance community preparedness. Strengthening disaster education and training is essential to ensure that the community possesses both the understanding and the skills necessary to handle emergency situations. In addition, the formation of community-based volunteer groups, especially in flood-prone areas, is essential to support the evacuation process, distribution of aid and dissemination of information when disasters occur. Collaboration between the community, government and social institutions also needs to be improved to ensure the effective distribution of information and resources. The implementation of technology-based early warning systems, such as mobile applications and short messages, must be developed to be easily accessible and used by the public. In addition, awareness of the importance of social capital, especially the value of trust and cooperation between citizens, needs to be increased as a foundation for strengthening community-based preparedness.

Further research directions are suggested to measure more specifically the direct impact of social capital on flood preparedness, including identifying key elements such as social trust, horizontal communication and community participation. The research must also analyse the differences in preparedness levels among regions – edges, middle and top – to tailor flood mitigation strategies to the specific characteristics of each local area. Furthermore, it is essential to evaluate the effectiveness of early warning technologies that have been or will be implemented by communities along rivers. This assessment will help determine the technology’s contribution to reducing the impact of flooding and enhancing community response. By integrating structural, social and technological approaches, the resilience of the communities along the Bengawan Solo River to flood disasters can be improved in a more comprehensive and sustainable manner.
